# 3D printed cellular solid outperforms traditional stochastic foam in long-term mechanical response

**DOI:** 10.1038/srep24871

**Published:** 2016-04-27

**Authors:** A. Maiti, W. Small, J. P. Lewicki, T. H. Weisgraber, E. B. Duoss, S. C. Chinn, M. A. Pearson, C. M. Spadaccini, R. S. Maxwell, T. S. Wilson

**Affiliations:** 1 Lawrence Livermore National Laboratory, Livermore, CA 94550, USA

## Abstract

3D printing of polymeric foams by direct-ink-write is a recent technological breakthrough that enables the creation of versatile compressible solids with programmable microstructure, customizable shapes, and tunable mechanical response including negative elastic modulus. However, in many applications the success of these 3D printed materials as a viable replacement for traditional stochastic foams critically depends on their mechanical performance and micro-architectural stability while deployed under long-term mechanical strain. To predict the long-term performance of the two types of foams we employed multi-year-long accelerated aging studies under compressive strain followed by a time-temperature-superposition analysis using a minimum-arc-length-based algorithm. The resulting master curves predict superior long-term performance of the 3D printed foam in terms of two different metrics, i.e., compression set and load retention. To gain deeper understanding, we imaged the microstructure of both foams using X-ray computed tomography, and performed finite-element analysis of the mechanical response within these microstructures. This indicates a wider stress variation in the stochastic foam with points of more extreme local stress as compared to the 3D printed material, which might explain the latter’s improved long-term stability and mechanical performance.

Cellular solids or foams^1^,^2^,^3^ are a very important class of materials with diverse applications ranging from thermal insulation and shock absorbing support cushions, to light-weight structural and floatation components, and constitute crucial components in a large number of industries including automotive, aerospace, electronics, marine, biomedical, packaging, and defense, just to name a few. In many of these applications the foam material is subjected to long periods of continuous stress, which can, over time, lead to a permanent change in structure and a degradation in performance^4^,^5^. Traditional foams are associated with non-uniform microstructures involving quasi-stochastic organization of materials and voids that involve significant dispersion in size, shape, thickness, connectedness, and topology (see Fig. 1(a)). Although, depending on the application, the overall porosity (and therefore density) and the average pore size can be controlled to some degree, the lack of control at the microstructural level makes it difficult to predict the long-term stability in structure and performance of such materials.

With the advent of 3D printing technology, also called additive manufacturing^6^, it is now possible to create uniform structures with well-defined cellular shapes and dimensions. Using a process called direct ink writing (DIW)^7^ the creation of additively manufactured (AM) foams was recently demonstrated^8^. The material is built up layer-by-layer, with each layer consisting of equally-spaced parallel cylinders of the same uniform diameter. Initially two simple architectures were considered: (1) a simple-cubic (SC) architecture defined by the layer arrangement ABABAB…, where cylinders in layers A are perpendicular to the cylinders in layer B; and (2) a face-centered tetragonal (FCT) architecture defined by the layer-arrangement A_1_B_1_A_2_B_2_A_1_B_1_A_2_B_2_…, where in addition to the cylinders in layers A_i_ and B_j_ (i, j = 1, 2) being mutually perpendicular, the layers in A_1_(B_1_) are shifted with respect to the layers in A_2_(B_2_) by half-a-pitch (see Fig. 1(b)). Additive manufacturing is well-suited to create complex 3D geometries without the need for expensive tools or fixtures, and with minimal post-processing^6^. Thus, one can easily imagine creating more complex chemical and topological variations of the AM foam structures mentioned above, with the promise of achieving interesting mechanical response properties, including negative modulus^9^, negative Poisson’s ratio^10^,^11^, and high modulus-to-weight ratios^12^,^13^. We should mention here that although all our designs so far have primarily involved woodpile structures, it is also possible to print other truly 3D structures. Such a project requires a multimaterial printing capability wherein a support material is co-printed with the material of interest. After printing, the support material is selectively removed, often through chemical or thermal means. The general approach is similar to one often employed in the popular 3D printing process called fused deposition modeling.

Although additive manufacturing can lead to engineering components with complex architecture and shape with high geometrical precision, one of the concerns, as with any new technology, is possible degradation in the long-term performance of AM parts. More specifically, for foam materials designed above, there is a critical need to compare the long-term performance behavior of the AM foam with that of traditional stochastic foam that the former seeks to replace. It is difficult to predict the outcome of such comparison beforehand because the difference between the foams is not only in the microstructure, but there are other fundamental differences as well. For instance, the constituent PDMS resins from which the stochastic and AM foams are created have filler particles of different chemical composition, sizes, and dispersion. Such differences are necessitated by the flow property requirements of the viscoelastic ink employed in the DIW technology.

In this paper, we report multi-year accelerated aging experiments on a stochastic PDMS foam and compare with a 12-month-long aging study on an AM FCT foam of comparable porosity. In all experiments the foam pads were subjected to a constant compressive strain. We focus on two long-term performance metrics, i.e., compression set and load retention, defined more precisely in the next section. We used a recently reported unsupervised time-temperature-superposition (TTS) algorithm to shift isotherms in the logarithmic time-axis to create master curves of these properties that can be directly compared between the two foams. To understand the differences in behavior of the two foams we analyzed X-ray computed tomography (CT) images of the foam microstructures under compression and performed finite-element calculations of the stress distribution around the pores.

## Accelerated Aging Studies and Time-Temperature Superposition

The stochastic foam sample used in the accelerated aging study was made by compounding a silica-reinforced PDMS resin with urea particles and curing at 121 °C for 2 hours in a mold. The urea was leached out with water and the foam post-cured at 204 °C for 18–24 hours, resulting in a stochastic foam 1.0 mm thick with ~63% porosity^14^,^15^. To print the AM foam, a commercially available silica-reinforced PDMS elastomer (Dow Corning SE 1700 clear adhesive) was used^7^. A 1.6 mm thick eight-layer structure was created with 250-μm-diameter strands, spaced to yield porosity comparable to the stochastic foam. Based on the manufacturer’s recommendation the AM FCT foam was cured at 150 °C for 1 hour.

Specimens were compressed in rigs comprised of two parallel steel plates bolted together with a given separation to achieve the desired compressive aging strain (25–35%) (see supplemental Fig. S1). Compressed specimens were aged at four different temperatures (room temperature, 35, 50, and 70 °C). Uncompressed specimen thickness and load at the aging strain were periodically measured using a load tester. Heated specimens were allowed to cool to room temperature under compression prior to measurement. With the bolts removed, the compression rig containing the specimen was positioned in the tester and load applied until the compression rig itself was under load, as indicated by a sudden change in slope of the load deflection curve (see supplemental Fig. S2).

The compression set (*S*(*t*)) is defined as the ratio of the decrease in sample thickness (after periodic removal of stress) at time *t* to the original engineering compression at time zero. In terms of the original specimen thickness *h*_*0*_ (before aging), the compressed thickness *h*_*c*_, and the uncompressed thickness (at time t) *h*_*t*_, it is given by:


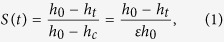


where *ε* is the engineering compressive strain (see Fig. S1). Load retention (*R*(*t*)) is defined by the ratio of the load at time *t* (F_*t*_) measured while the specimen is under the long-term compressive strain (during aging) to the corresponding load at time zero (*F*_*0*_) at the beginning of the aging study, i.e.,


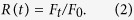


The above definitions of *S*(*t*) and *R*(*t*) make them relatively insensitive to the level of long-term strain employed, and thus make them comparable across all our experiments where the strain level varies between 25–35%. Note that both definitions above are properly normalized, i.e., compression set *S*(*t*) starts out with a value of 0% and increases toward a theoretical maximum value of 100%, which indicates complete loss of functionality. It is exactly the opposite for load retention, which starts out at an initial value of 100% and decreases monotonically toward a theoretical minimum of 0%, which represents complete lack of mechanical response. Conditions and parameters for the aging studies on different foams are summarized in supplemental Table 1. It is to be noted that compression set and load retention are not completely uncorrelated quantities – higher compression set is usually associated with lower load retention, given that the former implies less amount of load needed to get back to the original strain level. Additionally, load retention also includes effects of evolution in mechanical modulus as a function of time. Thus, together *S*(*t*) and *R*(*t*) provide a good description of the mechanical response state of the material and constitute good indicators of performance as a function of time. As far as measurement errors in our experiments, we estimate errors in thickness measurements to be less than 0.2% and errors in force measurements to be within 3%. Thus, all our compression set and load retention results reported below are accurate to within a few percent.

**Figure 2:**
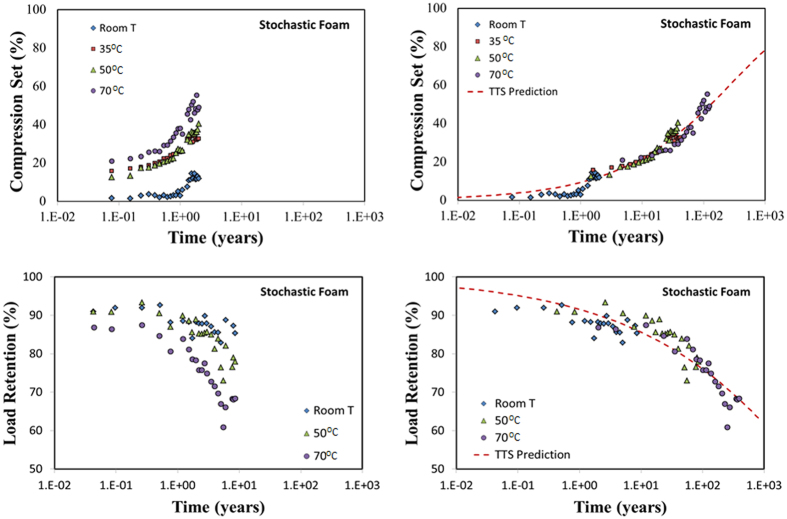
Compression set (top left) and load retention (bottom left) of an open-cell stochastic foam made from a PDMS elastomer. The left figures are actual measurements taken as a function of time over a period of 2 years for compression set and 8.5 years for load retention. The right figures are obtained by TTS-shifting the isotherms along the log-time axis so as to obtain a single master-curve with the minimum arc-length^21^. The dashed curves (TTS Prediction) are smooth fits to the master curve, and used for prediction purposes.

Figure 2 (top left) displays the compression set of the stochastic foam measured over a period of two years at four different temperatures: room temperature (i.e. ambient conditions), 35, 50, and 70 °C. In order to predict the long-time evolution of the compression set under ambient conditions, we performed a procedure called time-temperature superposition (TTS)^16^,^17^, in which each isotherm is rigidly shifted along the logarithmic time axis so as to generate a single “master” curve. In the literature one often encounters examples, especially on thermo-rheological response of polymers and composites, where such curves are manually shifted “by eye”. Although such manual shifting is acceptable for properties that can be accurately measured with little noise, in many cases such a procedure often can be subjective^18^,^19^ and may lead to large errors in long-term prediction. Given that the present work involves comparison of measurements on two different materials conducted over vastly different time-durations, a more accurate and objective method was necessary. To this end, we employed a recently developed geometry-based algorithm of TTS shifting^20^,^21^, in which the optimum master curve is defined as the one corresponding to the minimum vertical arc-length, given by the formula:


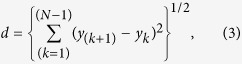


where 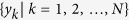 represent all observations at all different temperatures that have been arranged in the ascending order of shifted times. A schematic representation of the minimization procedure is provided in supplemental Fig. S3. More details are described elsewhere^21^.

Figure 2 (top right) shows the optimized master curve for the compression set data of Fig. 2 (top left) obtained by following the above TTS procedure. For the purpose of comparison between different foams, we also provide a smooth prediction curve defined by the three-parameter function:


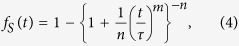


where the parameters *m*, *n*, and τ are obtained by minimizing the mean-square vertical deviation of the data points in the master curve from the prediction curve, and in the plot we multiply the function *f*_*S*_(*t*) by 100 and express as percent (%).

Figure 2 (bottom left) displays the measured load retention of our stochastic foam with data taken over a period of 8.5 years at room temperature, 50, and 70 °C, while Fig. 2 (bottom right) shows the master curve formed by the TTS-shifted data along with a smooth prediction master curve defined by the function:


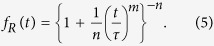


Data collection over a much longer time for load retention was necessitated by the requirement of property prediction over a period of several decades, in line with extended service times in many applications involving structural support foams.

**Figure 3:**
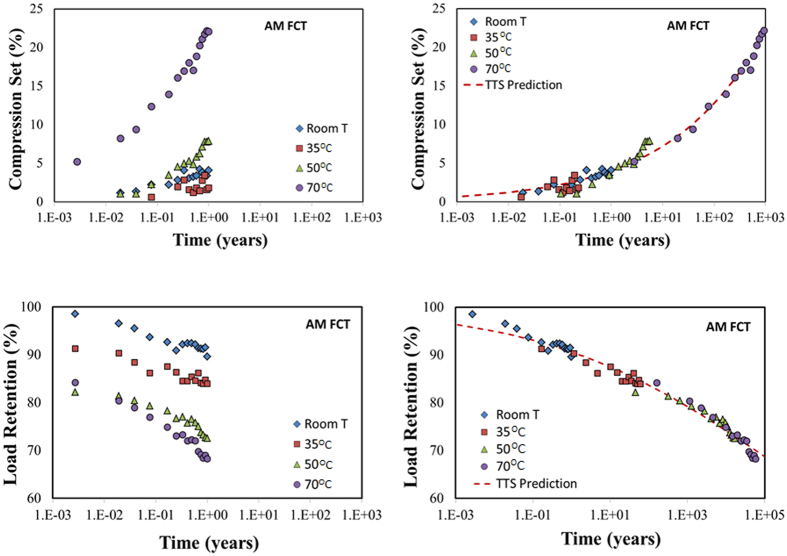
Compression set (top left) and load retention (bottom left) of AM FCT–a 3D printed PDMS foam of the face-centered tetragonal structure. The left figures are actual measurements taken as a function of time over a period of 1 year. The right figures are obtained by TTS-shifting the isotherms along the log-time axis so as to obtain a single master-curve with the minimum arc-length^21^. The dashed curves (TTS Prediction) are smooth fits to the master curve, and used for prediction purposes.

Figure 3 displays the results of compression set and load retention for the AM FCT foam (averaged over two samples) with data collected over a period of 12 months, along with the corresponding TTS-shifted master curves. Fortunately, the master curves in this case cover the required 100-year period, and we stopped collecting data at longer times.

**Figure 4:**
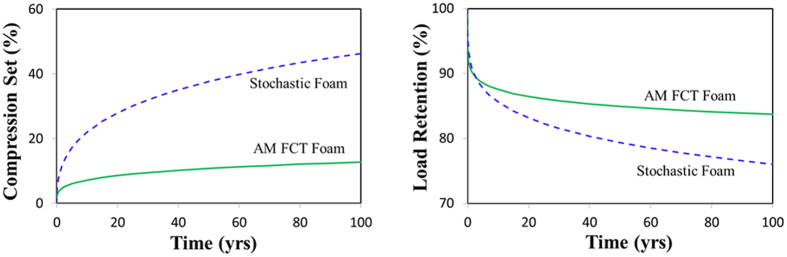
TTS-predicted compression set (left figure) and load retention (right figure) for stochastic and AM FCT foams under ambient conditions over a period of 100 years. The AM foam is clearly superior in both properties, except for load retention at very early times.

Figure 4 (top) compares the prediction curves for compression set and load retention for the stochastic and AM FCT foam materials over a period of 100 years (plotted in linear time-axis). From these curves it is clear that the AM FCT foam has clearly superior performance as compared to the stochastic foam, both in terms of compression set and load retention, except perhaps load retention during the first few years.

In order to gain some insights into the origin of the significant difference in the long-term behavior of the two foams, especially in terms of compression set, we first wanted to determine if there are intrinsic differences in the aging properties of the two rubber materials from which the foams are synthesized. To this end, we performed a short-term aging study on the respective rubber materials where cylindrical rubber pads were subjected to compressive strains of ~25%. In this test four samples of each rubber were subjected to 70 hour-long exposure times at 70 °C while under constant compression, and the compression set determined at the end of the 70 hour period. As Table 1 shows, the compression set in the rubber used with the stochastic foam is three times smaller than that in the rubber used with the AM foam. In the light of this result, the superior long-term performance characteristics of the AM foam can only be traced to its microstructural differences with its stochastic counterpart.

## X-Ray CT of Foam Microstructures

**Figure 5:**
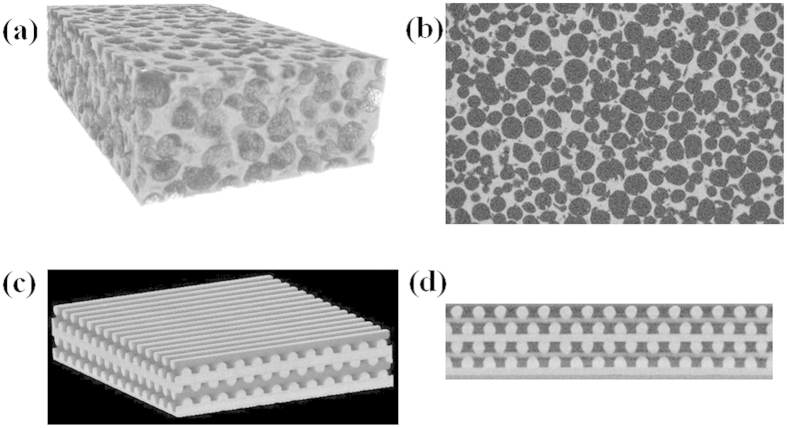
X-ray CT images of stochastic foam (**a**,**b**) and AM FCT foam (**c,d**) made out of PDMS elastomer. (**a**) 3D image of the outer surface of a rectangular specimen of a stochastic foam; (**b**) 2D image of a typical cross-section of the stochastic foam specimen; (**c,d**) side-top and end-cross-section views of a 8-layer AM FCT foam sample. Mesh representation of such images are used to perform finite-element simulation of stress distribution reported in this work.

In order to characterize and compare the microstructures of the two foams, we performed a series of X-ray CT analyses^22^,^23^,^24^,^25^,^26^. All scans were carried out using an Xradia system with a 0.5X objective at 70 kV and 10 W with a 1.25 s acquisition time. Reconstructed images were obtained with a voxel size of 14.7 microns. A custom built, in-house designed modular fixture was used as an in-situ sample rig in all X-ray imaging studies reported here. This fixture is based upon an original design by Maxwell, Chinn and co-workers^27^. The rig is designed such that one to five 15 mm diameter foam pads separated by Mylar or polyester shims can be compressed to a maximum of 50% of their original ~1 mm thickness in an inert, humidity controlled atmosphere. The compression rig is constructed from the polymer Delrin and the desiccant employed is a zeolite with molecular sieve size of ~4 Å. Multiple shims could be employed to achieve the desired compression ratio for a given set of samples. A yielding leaf spring inserted into the rig provides a load correction factor and conditions of constant load, regardless of dimensional change in the samples. Imaging was carried out both with and without load for all samples. Figure 5 displays typical X-ray CT images of the stochastic foam and AM FCT foam, both a 3D image of a rectangular sample and a 2D scan of a cross-section.

## Finite-Element Analysis of The Local Stress Distribution

The geometries and meshes of the stochastic and additively manufactured foams were generated using two different approaches. For the stochastic foam, a solid region with a square cross section was first extracted from the X-ray CT data using the cropping, segmentation, and smoothing capabilities in ScanIP from Simpleware^28^. A tetrahedral conformal mesh was then produced from the voxel data based on a variant of the marching cube method. The resulting mesh was verified to have the same known porosity of the stochastic foam. To match this porosity for the FCT architectures, we created a domain consisting of 8 layers of *D* = 250 μm diameter filaments with a pitch of 605 μm and an interlayer center-to-center spacing of 0.85 *D*. An automated scripting tool assembled the geometry and tetrahedral mesh. Two thin stiff plates bounded the vertical extents of the two foams and provided a means of compressing the upper surface through a linear displacement while constraining the lower surface. The materials model for both structures was derived from experimental compression measurements of a cylindrical specimen of the bulk rubber (SE 1700) used to synthesize the AM foam. The stress response curve was fit to a Mooney-Rivlin constitutive equation^29^ using a global optimization method to determine the coefficients. Quasi-static finite element simulations using the NIKE implicit code^30^,^31^ produced the overall mechanical response and local stress contours within the domains.

**Figure 6:**
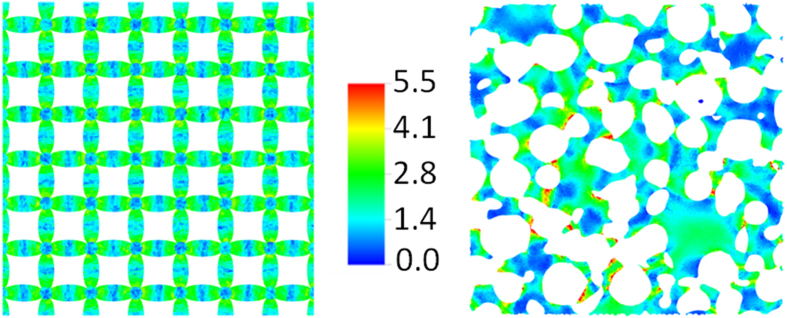
Stress distribution in a typical slice of: (left) AM FCT foam; and (right) stochastic foam, both under 15% compressive strain. The presence of many high stress points is clearly evident in the stochastic foam. The presence of these high-stress points over an extended period of time is likely responsible for higher compression set and lower load retention in the stochastic foam as compared to the AM FCT foam. The stress scale bar is in units of 10^5^ Pa.

Figure 6 shows the von Mises stress magnitude^2^ at representative lateral cross-sections of both architectures at 15% strain. The FCT plane intersects the overlap region between layers so filaments in both directions are visible. The direct ink write foam clearly exhibits uniform pore size and spacing whereas the stochastic foam contains a distribution of pore sizes with several in close contact. This clustering and overlapping of pores produces thin walls and highly concave topologies resulting in local stress concentrations indicated by the yellow and red regions in the figure. These points of high stresses are possibly the driving force behind irreversible damage to the foam microstructure, including strut fracture and pore collapse. On the other hand, consistent with its uniform architecture, the AM FCT foam exhibits highly repeatable and more uniform stress contours in both filament orientations with magnitudes less than a factor of two below the maximum stress in the stochastic foam.

## Discussion and Summary

While additive manufacturing continues to open up exciting materials design and application possibilities across diverse disciplines, there is a crucial need to address the long-term stability and performance of the AM parts and devices. This paper represents the very first study of this nature. Here the long-term mechanical characteristics of a 3D printed polymer foam is carefully compared with that of a traditional stochastic foam through the analysis of multi-year-long accelerated aging data using a time-temperature-superposition procedure based on geometric arc-length minimization. The resulting master curves predict clearly superior long-term performance of the AM foam, both in terms of compression set and load retention. This result is remarkable given that the AM foam is created out of rubber with three times the stronger propensity for permanent deformation as compared to the rubber constituting the stochastic foam. To gain insight, we have imaged the microstructures of both foams with X-ray computed tomography and carried out Finite-element analysis of stress distribution. Such analysis leads us to conclude that the superior long-term behavior of the AM foam is due to a more uniform local stress distribution pattern relative to the stochastic foam, which develops more extreme stress points within its microstructures. The latter is likely responsible for irreversible damage to the foam structure including pore collapse, strut fracture, and permanent deformation of the cell wall. Finally, we would like to point out that that the results presented here compares the aging of a stochastic foam with that of an AM foam of a very specific architecture. We acknowledge that there may be better performing AM designs, including other optimal 3D designs that we are yet to explore. Identifying and testing such novel micro-architectural designs is an area of future work.

## Additional Information

**How to cite this article**: Maiti, A. *et al.* 3D printed cellular solid outperforms traditional stochastic foam in long-term mechanical response. *Sci. Rep.* **6**, 24871; doi: 10.1038/srep24871 (2016).

## Supplementary Material

Supplementary Information

## Figures and Tables

**Figure 1:**
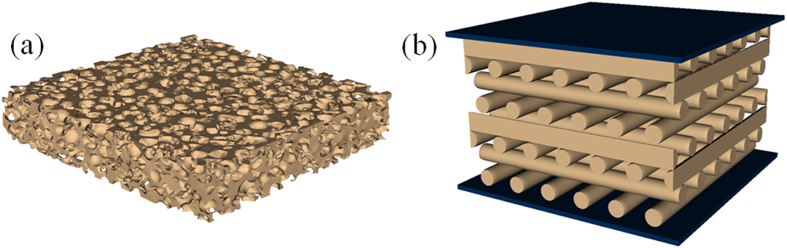
Microstructures of two different foam materials made out of filled polydimethylsiloxane (PDMS) elastomers: (**a**) an open-cell stochastic foam; and (**b**) an additively manufactured (AM) foam with the face-centered-tetragonal (FCT) lattice structure, the diameter of each cylindrical strut being 250 μm.

**Table 1:** Short-term accelerated aging study (70° C for 70 h) of the Compression Set of the rubber materials constituting the two types of foams.

Material	Specimen #	Original Thickness(mm)	CompressiveStrain (%)	Final Thickness(mm)	CompressionSet (%)
Rubber used forAM FCT foam	1	12.97	26.7	12.76	6.1
	2	12.98	26.8	12.78	5.9
	3	12.97	26.7	12.76	5.9
	4	12.95	26.6	12.74	6.0
	Avg ± SD				6.0 ± 0.1
Rubber used forstochastic foam	1	12.64	24.8	12.58	2.0
	2	12.68	25.0	12.59	2.8
	3	12.64	24.8	12.60	1.2
	4	12.68	25.0	12.63	1.5
	Avg ± SD				1.9 ± 0.7
