# Corrigendum: 3D printed cellular solid outperforms traditional stochastic foam in long-term mechanical response

**DOI:** 10.1038/srep26573

**Published:** 2016-05-25

**Authors:** A. Maiti, W. Small, J. P. Lewicki, T. H. Weisgraber, E. B. Duoss, S. C. Chinn, M. A. Pearson, C. M. Spadaccini, R. S. Maxwell, T. S. Wilson

Scientific Reports 6: Article number: 2487110.1038/srep24871; Published online: 04272016; Updated 05252016

The Acknowledgements section in this Article is incomplete.

“We would like to sincerely thank Dr. Jim Schneider of National Security Campus, MO (formerly Kansas City Plant) for giving us access to the results of their load retention study on the stochastic foam material. This work was performed under the auspices of the US. Department of Energy by Lawrence Livermore National Laboratory under Contract DE-AC52-07NA27344”.

should read:

“We would like to sincerely thank Dr. Jim Schneider of National Security Campus, MO (formerly Kansas City Plant) for giving us access to the results of their load retention study on the stochastic foam material. The authors would also like to gratefully acknowledge Dr Jessica Maisano and the University of Texas High-Resolution X-ray CT Facility for performing all CT scanning of the materials presented here. This work was performed under the auspices of the US. Department of Energy by Lawrence Livermore National Laboratory under Contract DE-AC52-07NA27344”.

