# Supplementary material for: 3D printed cellular solid outperforms traditional stochastic foam in long-term mechanical response
Source: Sci Rep. 2016 Apr 27;6:24871. doi: 10.1038/srep24871 (PMC4846814; doi:10.1038/srep24871)
Supplement: Supplementary Information [file srep24871-s1.pdf]

Supplemental information:

Paper: "3D printed cellular solid outperforms traditional stochastic foam in long-term mechanical response," by A. Maiti, W. Small, J. Lewicki, T. H. Weisgraber, E. B. Duoss, S. C. Chinn, M. A. Pearson, C. M. Spadaccini, R. S. Maxwell, and T. S. Wilson

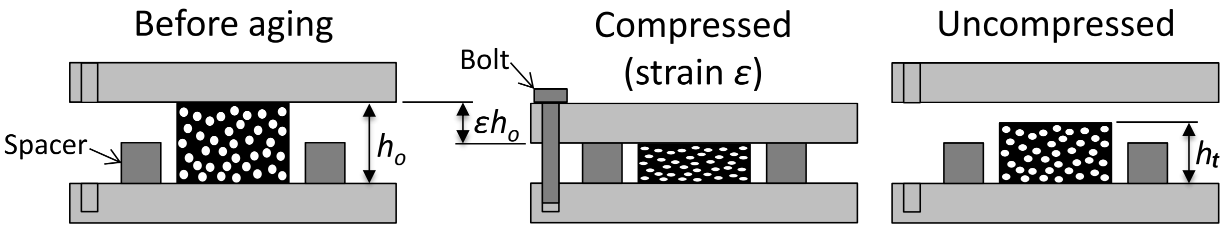

Fig. S.1. Schematic showing the various measured thicknesses used to define compression set: $S(t) = \frac{h_0 - h_t}{\varepsilon h_0}$

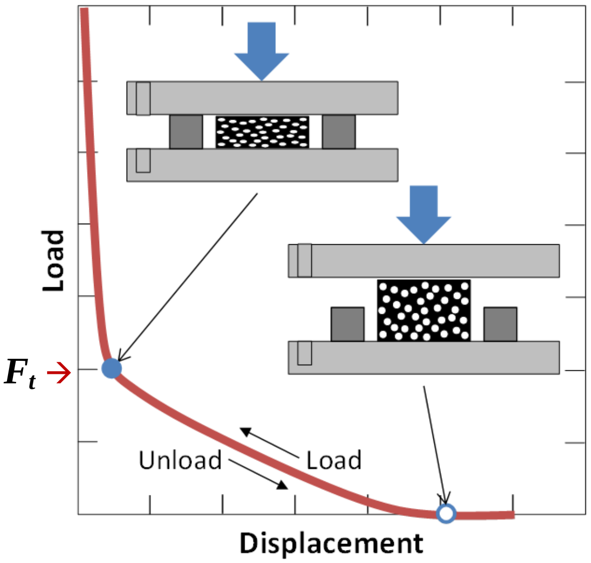

Fig. S.2. Schematic showing the measurement of force (load) under compression that is used to define load retention: $R(t) = F_t/F_0$; F_0 is the measured value of load at time zero, i.e. at the beginning of aging, while F_t is the measured value of load at time t. The force measurements are made at the bend in the load curve right before the sharp upward increase due to the compression of the rig blocks (closed dot). The difference between the displacement at the open dot (where the load begins to increase as the sample starts to undergo compression) and closed dot added to the spacer thickness gives the sample thickness used to calculate compression set.

Note: Though not expected to significantly affect the results, we should point out that the time zero values of h_0 and F_0 for the heated AM FCT foam samples were determined in a slightly different manner than those for the room temperature samples. The value of h_0 was obtained from the load deflection curve (see Fig. S.2) given by the load tester for the room temperature samples, while a digital thickness gauge was used for the heated samples. The digital thickness gauge values were reduced by an offset of 0.012 mm (determined experimentally) to compensate for the slightly higher measurement force in the load tester (8.5 kPa) relative to the thickness gauge (7.9 kPa). Unlike the values of F_0 for the room temperature samples which were obtained from their load deflection curves (see Fig. S.2), those for the heated samples were estimated using the load deflection curve obtained from a separate representative sample with the same microstructure made using the same DIW process and curing profile.

S. Table 1: Conditions and parameters for accelerated aging studies on different foams considered in our study.

	AM FCT Foam Study	Stochastic Foam Studies	
Values Measured	Compression set and load retention (single study)	Compression set	Load retention
Study Duration	1 year	2 years	8.5 years
Aging Atmosphere	Dry nitrogen	Dry nitrogen	Air (30-60% relative humidity)
Specimen Thickness	1.6 mm	1.0 mm*	1.0 mm
Aging Strain	30%	25%	35%
Aging Temperatures	Room T, 35, 50, 70°C	Room T, 35, 50, 70°C	Room T, 50, 70°C

*Three specimens were stacked in each compression rig, separated by thin metal shims to prevent inter-digitation of the foam surfaces; all calculations accounted for the metal shim thickness.

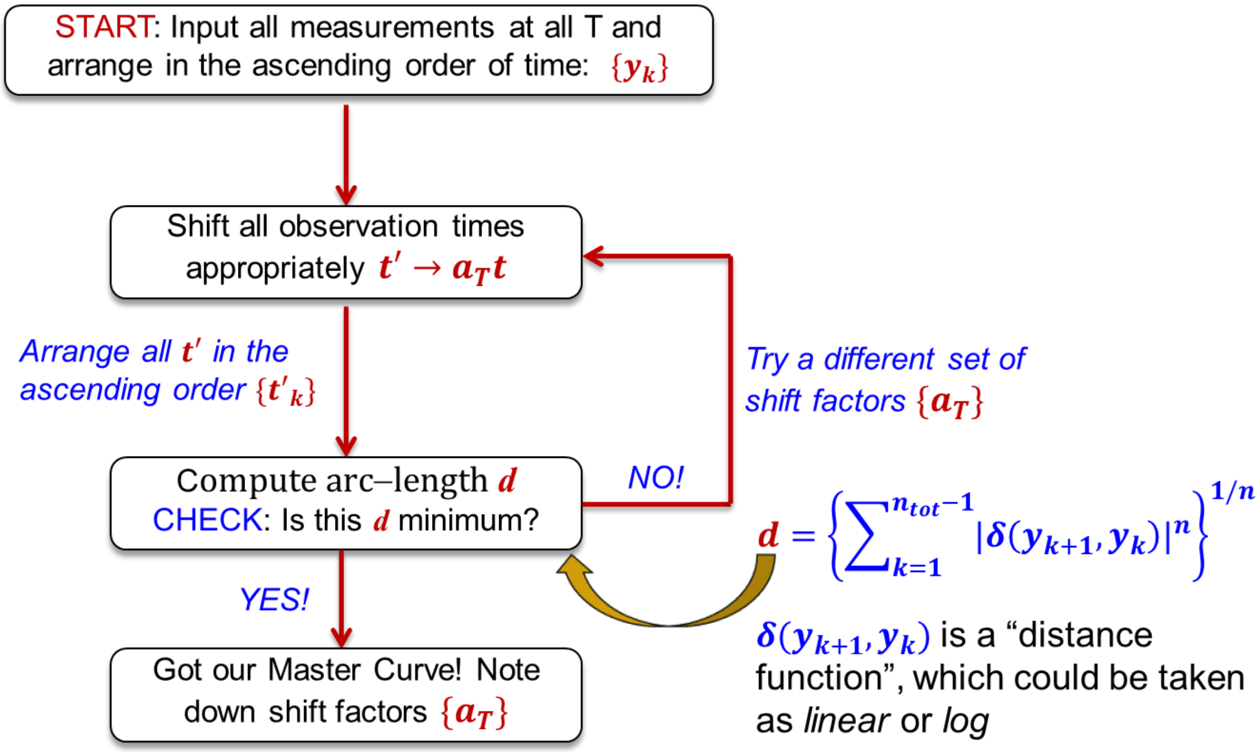

Fig. S.3. Schematic showing the iterative procedure by which the minimum-arc-length master curve is obtained in our time-temperature-superposition approach.
